# Stage 2 Registered Report: There is no appreciable relationship between strength of hand preference and language ability in 6- to 7-year-old children

**DOI:** 10.12688/wellcomeopenres.15254.1

**Published:** 2019-05-13

**Authors:** Verena E. Pritchard, Stephanie A. Malone, Kelly Burgoyne, Michelle Heron-Delaney, Dorothy V.M. Bishop, Charles Hulme

**Affiliations:** 1 Institute for Learning Sciences & Teacher Education, Australian Catholic University, Brisbane, Queensland, Australia; 2 Division of Human Communication, Development and Hearing, University of Manchester, Manchester, UK; 3 Department of Experimental Psychology, University of Oxford, Oxford, Oxfordshire, UK; 4 Department of Education, University of Oxford, Oxford, Oxfordshire, OX2 6PY, UK

**Keywords:** hand preference, laterality, language, development

## Abstract

**Background:** Weak or inconsistent hand preference has been postulated to be a risk factor for developmental language delay. Following on from our Registered Stage 1 report this study assessed the extent to which variations in language skills are associated with the strength of hand preference.

**Methods:** Data are drawn from a large sample ( *N* = 569) of 6- to 7-year-old children unselected for ability, assessed at two time points, 6 months apart. Hand preference was assessed using the Quantitative Hand Preference (QHP) task and five uni-manual motor tasks. Language skills (expressive and receptive vocabulary, receptive grammar, and morphological awareness) were assessed with standardized measures.

**Results:** We found QHP scores did not distinguish children with weaker language skills from those with stronger language skills and the correlation between QHP scores and language ability was negligible in this study. Hand preference on the QHP task was significantly stronger among right-handed than left-handed children and left-handed children were typically inconsistent in the hand used across different tasks.

**Conclusions: **The findings presented here fail to provide any support for the theory that weak cerebral lateralisation (as assessed here by the QHP task) places children at risk of language difficulties **. **

**Stage 1 report:** https://doi.org/10.12688/wellcomeopenres.15077.1

## Relationships between handedness and language skills in children

Humans typically show cerebral lateralisation for language. For the majority of the population, language processing appears to depend predominantly on left hemisphere systems. Much of the early evidence for cerebral lateralisation came from studies of adult stroke patients which showed that damage to the left hemisphere is more commonly associated with language deficits than damage to the right hemisphere ( Benson & Ardila, 1996; Damasio, 1992; Geschwind, 1971; Kertesz & McCabe, 1977). Patterns of cerebral lateralisation for language are also associated with measures of hand preference/hand function such that most adults with left-hemisphere dominance for language also show greater dexterity with the right hand ( Khedr *et al.*, 2002; Knecht *et al.*, 2000; cf. Mazoyer *et al.*, 2014). This has led to the use of handedness as a marker for the cerebral lateralisation of language. Evidence shows that left-handers are indeed more likely than right-handers to have atypical lateralisation for language ( Knecht *et al.*, 2000; Szaflarski *et al.*, 2002); around 30% of left-handers versus 5% of right-handers have atypical lateralisation for speech.

Studies of cerebral lateralisation in adults lead naturally to ideas about possible links between the development of cerebral lateralisation and language skills in children. If the development of cerebral lateralisation is critical for the development of language, handedness (as a proxy for the cerebral lateralisation of language) might be expected to relate to developmental language difficulties ( Annett, 2002; Bishop, 2013; Bishop *et al.*, 2014). In line with this, in some studies right-handedness has been reported to be associated with better language and literacy skills (see Somers *et al.*, 2015 for a review). However, evidence for such associations is mixed. In their meta-analysis which included studies of both children and adults, Somers *et al.* (2015) found no overall difference in verbal ability between right- and left-handed people (Hedges’ *g* = −0.03, *p* = 0.22). A follow up analysis of studies that included only children reported a very small effect favouring right-handed children (Hedges’ *g* = −0.09) though this effect was reduced to nonsignificant levels after excluding two studies with disproportionately large sample sizes (Hedges’ *g* = −0.06). It seems clear from the Somers *et al.* meta-analyses that any association between hand preference (treated as a binary variable; left versus right) and language ability in the general population is trivial in size, irrespective of age.

The absence of any clear relationship between handedness and language skill in the Somers *et al.* (2015) meta-analysis might reflect the fact that manual laterality is at best a weak proxy for language lateralisation in the brain ( Groen *et al.*, 2013). In response to this, some studies have used physiological measures of laterality (e.g., functional Transcranial Doppler Ultrasound). Some of these studies have found evidence for differences in language laterality in children with language difficulties (Developmental Language Disorder (DLD); see Wilson & Bishop, 2018 for a review). However, in the largest study of this sort Wilson and Bishop failed to replicate such an association and current evidence suggests that there is little, if any, relationship between physiological measures of language laterality and language skills.

There is, however, a more nuanced view of the possible relationship between laterality and language skill that needs to be considered. In general, individual differences in laterality are seen as stable characteristics, but there is also evidence that some aspects of laterality mature with age: children progress from a rather ambivalently expressed hand preference to a more consistent hand preference ( Scharoun & Bryden, 2014). In this view, the delayed development of cerebral lateralisation may be associated with language difficulties. This implies that children who show weak or inconsistent hand preference may be at risk for developmental language disorder (DLD). Note that on this view, left- versus right-handedness is not expected to be associated with language status: the crucial aspect is not the direction of laterality, but rather the consistency of that lateralisation, whether to left or right.

To assess this idea, Bishop and colleagues ( Bishop *et al.*, 1996) developed a measure of quantitative hand preference (QHP). In the QHP task the person stands in front of a table with a set of cards arranged on either side of the midline. The task is simply to pick up cards one at time (in response to a verbal command signifying the picture on the card) and place them in a box at the midline. This task gives a simple quantitative measure of hand preference (the proportion of cards picked up with the right hand) on a task that appears to have minimal cultural influence. Using the QHP task, Hill & Bishop (1998) reported that 7- to 11-year-old children with DLD and children with developmental coordination disorder showed less clearly defined hand preference on the QHP task than age-matched controls (but similar levels of hand preference to a younger control group who were roughly 3 years younger). Because performance on the QHP task was impaired in both children with language and motor disorders, Hill and Bishop concluded that the “QHP task appears to be a sensitive, but non-specific, indicator of developmental disorders” (p. 295). A note of caution is needed however, since the group sizes in the Hill and Bishop study were small (12 children with motor difficulties, 20 children with language difficulties and 26 age-matched controls). However, other studies by Bishop and colleagues that have used the QHP task with larger samples (i.e., Bishop, 2001; Bishop, 2005; Hill & Bishop, 1998) do indicate that the QHP task is generally more successful than traditional hand preference inventories such as the Edinburgh Handedness Inventory ( Oldfield, 1971) in differentiating between children with specific language impairment and age matched controls.

Although the Wilson & Bishop (2018) findings are not encouraging, a stronger test of a maturational hypothesis would involve testing children's consistency of lateralisation at two ages. We would predict that we should see a shift from less consistent to more consistent lateralisation over time, and that earlier establishment of consistent hand preference would be correlated with better language skills.

If weak cerebral lateralisation (as assessed by the QHP task) is a risk factor for language difficulties, it should be possible to detect such effects in large representative samples of children. Within such samples, children with the weakest language skills should be expected to show evidence of less clearly defined hand preference on the QHP task. In the current study we use observations of hand preference and the QHP task with a large unselected sample of children seen on two occasions. Analyses were performed treating language as a continuously distributed trait, as well as examining whether measures of handedness differ between children with particularly poor language skills versus controls.

## Methods

### Ethical considerations

The Australian Catholic University Human Research Ethics Committee provided ethical approval (2015-269H). Informed written consent was sought from the principals of the schools involved for all children enrolled in their first year of school (Preparatory Year) in January 2016. An opt-out procedure was followed. Parent information leaflets and opt-out consent forms were distributed to the parents of enrolled children (via both electronic and written hard copy format for each participant). All children in each class participated in the study unless parents signed the opt-out consent form for their child before the study’s commencement date.

### Participants

A total of 569 children from 11 schools in Brisbane, Australia participated as part of a larger longitudinal study ( Burgoyne *et al.*, submitted; Malone *et al.*, 2019). The sample size was determined largely by constraints on funding. We recruited the largest sample that we could given the staffing levels available. The sample size is very large for a longitudinal study of cognitive development using individually administered measures. The schools selected are essentially a convenience sample and consist of a sub-sample of the schools located in the greater Brisbane area who were approached with a request to participate. According to Government data on the socio-economic composition of the population in each school (the Index of Community Socio-Educational Advantage; ICSEA) eight of the participating schools serve a student population with an average level of educational advantage (ICSEA values between 997 and 1090 where the average range (1 *SD* of the mean) is 900 to 1100). The three remaining schools have higher ICSEA values (1112–1153) reflecting a student population with slightly above average levels of social advantage. Children were assessed at two time points: Within the final half of Year 1 (time 4 *n* = 496; mean age 81.23 months; range 71–99 months), and again approximately 6 months later during the first half of Year 2 (time 5; *n* = 454; mean age 87.74 months; range 77–106 months).

### Measures and procedure

As part of the larger longitudinal study ( Burgoyne *et al.*, submitted; Malone *et al.*, 2019), children completed a battery of scholastic, cognitive and motor measures at time 4 and time 5 including measures of language and hand preference. All measures were administered individually in the children’s schools by two of the authors (SM; VP) and four postgraduate research assistants.

### Handedness

**Figure 1.**
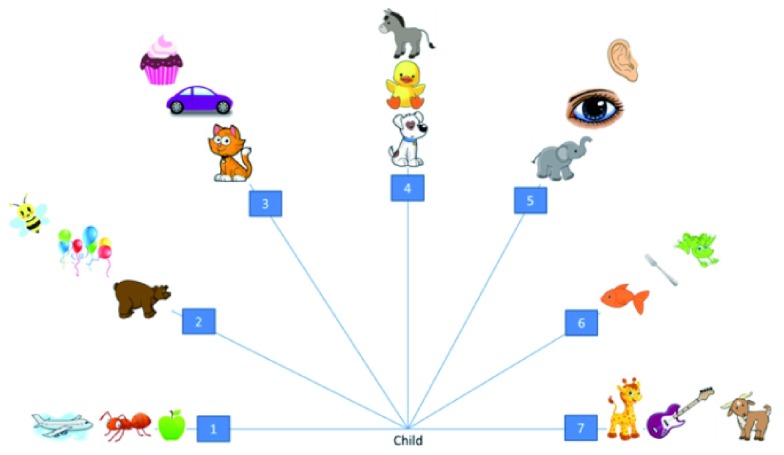
Illustration of the items and spatial positions in the Quantitative Hand Preference (QHP) task.

***Quantitative hand preference (QHP) task.*** Bishop *et al.*’s (1996) QHP task was used to quantify the degree of hand preference. For this task children were required to stand and reach for individual picture cards one at a time placed on a waist-high table. The cards were positioned at one of seven positions extending at 30 degree intervals from the left to the right of the child’s midline to form a semi-circle. There are 21 trials in total (three cards spaced along each of the seven positions of which there are three to the left, one at midline, and three to the right). Children were asked to stand at the midline position and to pick up a named card and place it into a box directly in front of them. Figure 1 shows the items and set-up for the QHP task. Card selection followed a fixed random order and no time constraints were imposed. Reaching was scored following Bishop, 2005: one point is awarded for each reach done with the right hand, 0 points for bimanual usage or unclear preference, and -1 point for each reach done with the left hand.

***Unimanual hand preference motor tasks.*** Measures of the hand used while children completed the following 5 motor tasks at both time 4 and time 5 were recorded. (1) *Lace Threading* (Movement Assessment Battery for Children – Second Edition (Movement ABC-2; Henderson *et al.*, 2007): this task requires children to thread a string through eight holes on a board – a practice trial of four holes is followed by two test trials; (2) The Movement ABC-2 *Drawing Trails* subtest. This requires children to trace a pattern with a pen between two lines without lifting the pen from the page or crossing the lines - one practice trial and two test trials were completed. 3) The *Apples Selection* task ( Breckenridge, 2008): this requires children to identify as many red apples as they can ( *n* = 30) printed on a page within a 60 second time limit while ignoring white distractor apples and red distractor strawberries. 4) The *Two-Match Shifting* task ( Dick, 2014): in this task children are presented with a series of three boxes containing picture arrays. These pictures varied from each other along four dimensions: 1) object (boat, rose, rabbit); 2) colour (red, green, blue); 3) size (small, medium large); 4) quantity (one, two, three), and for each test trial children were asked to point to two pictures in one box that were the same as each other on one dimension but different from the pictures in the other box. For each test trial, there were two possible ways that the boxes could match - children completed 12 test trials. 5) *Token Placing* ( Cohen, 1997): in this task children are shown a 4 × 4 grid with a pattern of 8 red dots for 5 seconds and asked to recreate it on an empty grid using plastic discs – five test trials were completed for one pattern, and one trial for a different pattern.

### Language

***Expressive vocabulary.*** The Expressive Vocabulary subtest from the CELF 4 ^AU^ ( Semel *et al.*, 2006) was used. In this test, children are asked to name a series of pictures depicting objects (e.g. skeleton, saxophone) or actions (e.g. drawing). Testing was discontinued after 7 consecutive errors. Each response is scored as 0 (incorrect), 1 (partial response) or 2 (correct response).

***Receptive vocabulary.*** The Receptive One-Word Picture Vocabulary Test ( Brownell, 2010) was used to assess children’s *Receptive Vocabulary*. For this test, children were presented with four pictures and asked to point to the picture that matched the word spoken by the examiner. All children started at the item corresponding to the 7.0–7.11 age bracket. A basal level was established by scoring eight consecutive correct responses, and a ceiling was established by scoring six incorrect responses within eight consecutive items. Testing was discontinued after the ceiling had been established. A score of 1 was awarded for each correct response.

***Receptive grammar*.** A shortened version of the Test for Reception of Grammar – Second Edition (TROG-2; Bishop, 2003) was used to assess children’s understanding of grammatical contrasts. The version of the TROG-2 used in the current study included 40 stimulus items arranged in blocks of 2, which test 20 grammatical contrasts (e.g. the prepositions “in” and “on”, pronouns, relative clauses). For each item, children were presented with a four-picture array (one target item and three distractor items including lexical and/or grammatical foils) and asked to point to the picture that best represents the grammatical or lexical element contained in the target sentence produced by the examiner.

***Morphological awareness.*** Children’s morphological awareness was assessed with a Word Analogy task ( Kirby *et al.*, 2012) that includes both inflectional and derivational transformations. In this task, children are/were asked to provide a missing word based on an analogical pattern for 10 inflectional items and 10 derivational items. For these, the experimenter would say a pair of words, for which the second word included a morphological shift. Then a target word was spoken and children were asked to apply the same morphological shift to this word as in the first pair (e.g., walker:walk::teacher: *teach* for inflection, and sleep:sleepy::cloud:: *cloudy* for derivation). A series of six practice items were provided first in which children were corrected if they gave an incorrect response. The child’s score was the total number of correct answers for both inflected and derived words.

## Analysis plan

**Figure 2.**
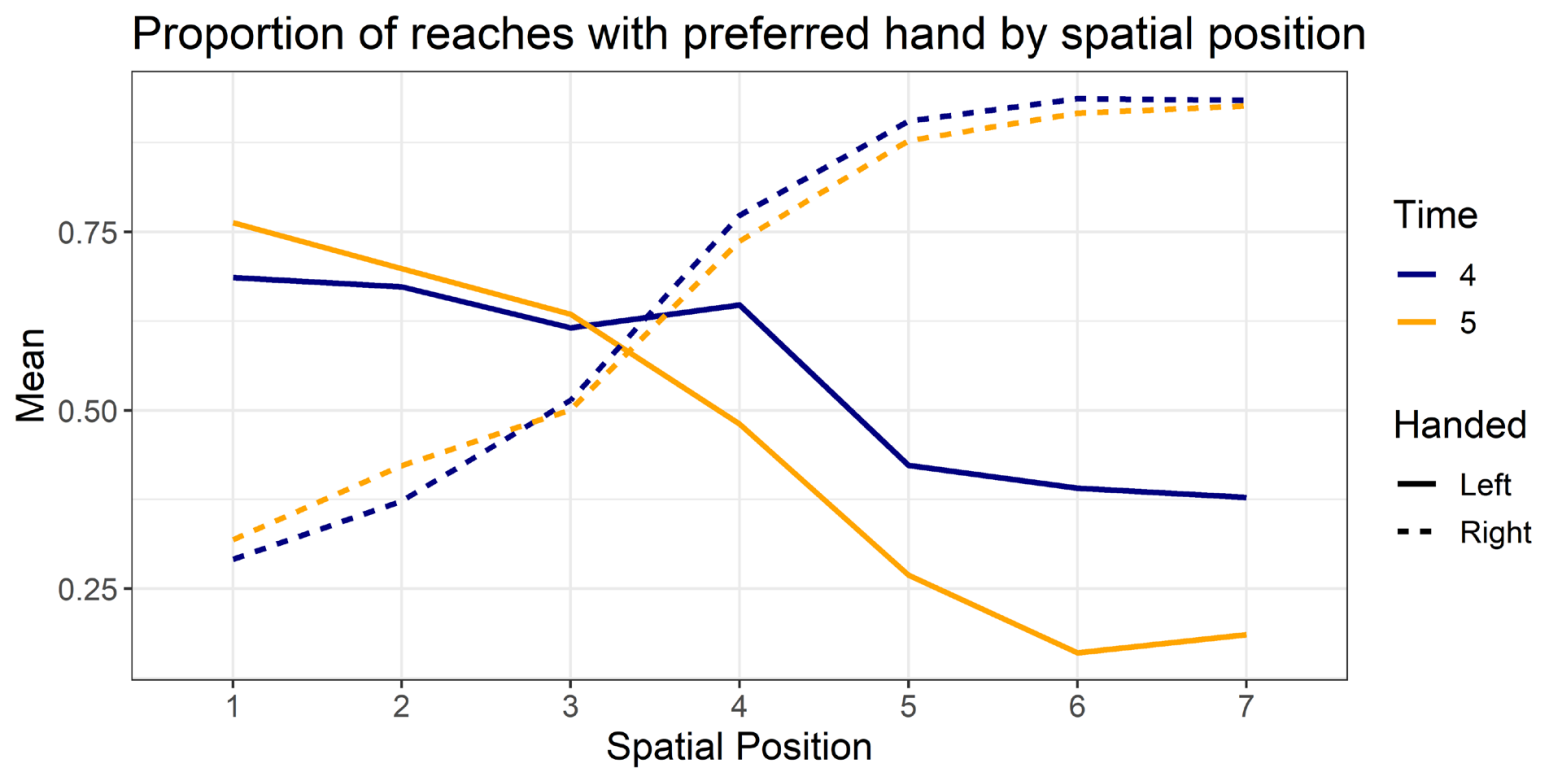
Proportion of reaches with the preferred hand in each of the seven spatial positions at time 4 and time 5 on the Quantitative Hand Preference (QHP) task.

**Figure 3.**
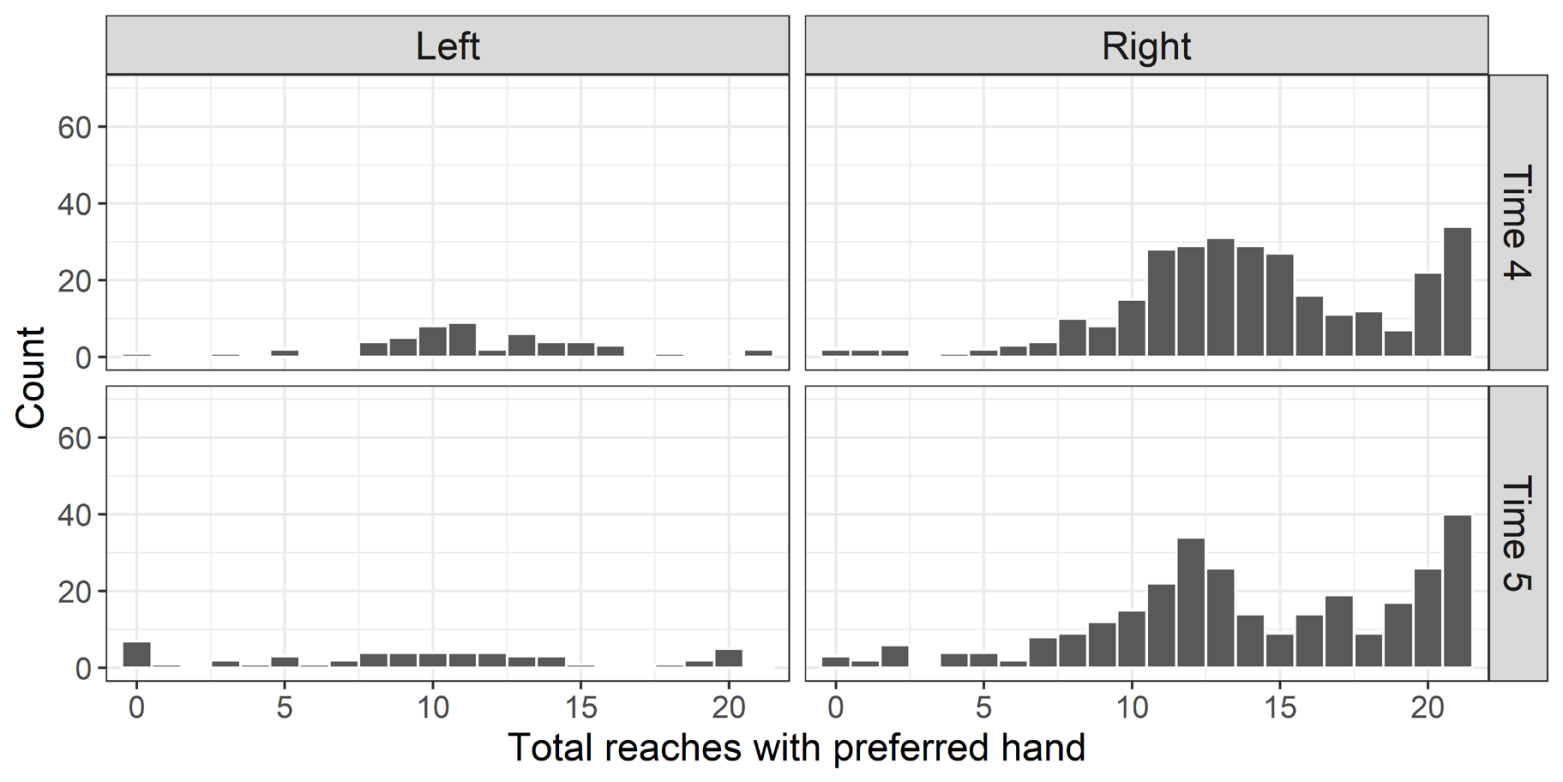
Histograms depicting the distribution of reaching scores for left- and right-handers on the Quantitative Hand Preference (QHP) task (time 4, upper panel, time 5, lower panel).

The majority of the analyses were conducted in Stata (v 15.1 StataCorp., 2017) though Figure 2 and Figure 3 were produced in R (v3.5.2). Our data are accessible on OSF (Underlying data ( Pritchard *et al.*, 2019)).

One complication in these data is that children can be either right or left handed. It can be expected that roughly 90% of the sample will be right-handed. The analysis was initially conducted on right- and left-handed children separately. We combined these samples if initial analyses of the separate groups support this. We have small amounts of missing data at each time point, and the data at time 5 is for a slightly reduced sample compared to time 4, details of how missing data were handled are given below.

We define handedness by the hand used to hold the pen in the Drawing Trails task.

We measured performance on the QHP task by the proportion of reaches made with the preferred hand.

We address a series of questions by performing the following analyses:

### Primary research questions – The reliability of the QHP task and the relationship between hand preference on the QHP and language ability

1. How reliable is the QHP task? We assessed the test-retest reliability of the QHP task. This is the correlation between the number of reaches with the preferred hand at time 4 and time 5. We computed three correlations: 1. For the right-handed sample; 2. For the left-handed sample; 3. For the combined sample.
2. Do children with language difficulties show weaker hand preference on the QHP task than children without language difficulties? We computed a language factor score based on the 4 measures of language ability that are available at both time 4 and time 5 (CELF Expressive Vocabulary; the Receptive One-word Picture Vocabulary Test; the Test for Reception of Grammar; and the Word Analogy task). We used independent sample t-tests to assess whether children with language difficulties (those with language factor scores less than or equal to 1 standard deviation below the mean) make a lower number of reaches with the preferred hand than children without language difficulties (the rest of the sample). We initially performed these tests separately for the time 4 and time 5 data and separately for left- versus right-handers (four separate independent samples t-tests). Because the patterns for left- and right-handers looked comparable we combined the two samples at each time point to give tests of greater statistical power.
3. In the population as a whole, do variations in language skills correlate with strength of hand preference on the QHP task? We assessed whether there is a relationship between language factor scores (treated as a continuous variable) and the number of reaches with the preferred hand on the QHP task. We performed linear regression analyses 1. For right-handers; 2. For left-handers and (assuming the relationships seem similar for the two samples); 3. For the combined sample. In all of these regression models we examined the adequacy of a linear model and checked for any undue influence of outliers.

### Secondary research questions – Possible developmental effects on the QHP task, and relationships between QHP performance and other measures of handedness

4. Does the strength of hand preference (number of reaches with the preferred hand) increase with age (is it higher at time 5 than time 4?). Any such increase could be taken as evidence of maturation or alternatively merely evidence of a practice effect. For children in the sample who were tested at both time points we computed 3 paired-samples *t*-tests comparing the mean number of reaches with the preferred hand 1. For the right-handed sample, 2. For the left-handed sample; 3. For the combined sample
5. Is the strength of hand preference (the number of reaches with the preferred hand) equivalent in right- and left-handers (or are left-handers less strongly lateralized?). We computed 2 independent samples t-tests comparing the proportion of reaches on the QHP task with the preferred hand in right- versus left- handers at both times of measurement (time 4 and time 5).
6. Do QHP scores differ for right-handed children with a consistent versus inconsistent hand preference as found by Bishop *et al.* (1996)? Consistency of hand preference here was defined by the 5 motor tasks described above; consistent hand use was defined as a child who uses the same hand for all 5 tasks. We computed 2 independent samples t-tests comparing the number of reaches with the preferred hand on the QHP in 1. consistent versus inconsistent right-handers and 2. consistent versus inconsistent left-handers (the sample size for left-handers will be small so the power in this latter analysis may be low).

### Study timeline

The dataset is a secondary registration of a pre-existing dataset. Data was collected across two time points separated by a six month interval: Time 4 in the last half of children’s second school year (August – December, 2017); Time 5 in the first half of their third school year (February – July, 2018).

## Results

### Patterns of missing data

We present analyses on a subset of data collected in a large scale longitudinal study ( *N* = 569 at time 1). At both time points considered here (time 4 and time 5) there were some missing data. Seventy children had missing data on all measures of handedness at both time points (i.e., *QHP task, Lace Threading, Drawing Trails, Apples Selection task, Two-Match Shifting task, Token Placing*), leaving a total initial study sample of *n* = 499. Missing data reflected absence from school, insufficient testing space to allow for the set-up of the QHP task or unfavourable environmental conditions if testing was conducted outside. Comparisons between the children with missing data on all handedness measures and those with data present at time 4 and time 5 showed that they did not differ from the complete sample in gender (χ2 = .321, *p* =.57) or age at time 1, *t*(1, 567) = -1.34, *p* = .19, *d* = .16)

For the children included in the handedness analyses, missing data were dealt with in the following ways: Of the 496 children assessed at time 4, the numbers completing the QHP and Drawing Trails tasks were 421 and 442, respectively. Of the 454 children assessed at time 5, 413 completed the QHP task and 426 completed the Drawing Trails tasks. In all analyses handedness was based on the hand used to hold a pen in the Drawing Trails task at a given time point. If the Drawing Trails task had not been administered, the child’s QHP score at the corresponding time point was coded as missing, since handedness could not be coded.

Analyses examining the test-retest reliability of the QHP task were restricted to those children who had handedness recorded on the Drawing Trails task at time 4 and time 5 and a QHP score recorded at both time points. For analyses of relationships between hand preference on the QHP task and language ability, factor scores for language at time 4 and at time 5 were created by running a Confirmatory Factor Analysis (CFA) in Stata 15 ( StataCorp., 2017); missing values for individual language measures at a given time point were handled using Stata’s mlmv estimator. Any children with missing values on all individual measures of language at either time point were coded as missing (total *n* = 51).

The critical variables entered into the following analyses are: time 4 Language Factor Scores, time 5 Language Factor Scores, time 4 QHP scores and time 5 QHP scores. We assessed whether patterns of missing data for these variables could be considered to be missing completely at random (MCAR) and they could (Little’s MCAR test, χ2 = 16.80, *p* =.27). The results of the analyses that we report below using pairwise deletion therefore should not be biased by patterns of missing data.

### Proportion of right-handed versus left-handed children

Children were classified as being left-handed or right-handed based on the hand they used to hold the pen in the Drawing Trails task at a given time (time 4 or time 5). At time 4, of the 442 children completing the Drawing Trails task, 46 (10%) were classified as left-handed and 396 (90%) as right-handed. Of the 426 children completing the Drawing Trails task at time 5, 61 (14%) were classified as left-handed and 365 (86%) as right-handed. Table 1 shows the age and gender distributions for these samples. Of the children for whom the Drawing Trails task data were available at both time points, 95% were consistent in their dominant hand across time points, while 5% changed hands (2 from left to right; 16 from right to left).

**Table 1.** Age (in months) and gender distributions for left- and right-handed children at time 4 and time 5.

Time point	Age: Mean ( *SD*)	Range	Gender: *n* (% male)
Time 4			
Left-handed ( *n* = 46)	80.11 (4.88)	73 – 93	27 (59)
Right-handed ( *n* =396)	81.28 (4.07)	71 – 99	184 (54)
Time 5			
Left-handed ( *n* = 61)	86. 79 (3.89)	81 – 98	34 (55)
Right-handed ( *n* =365)	87. 84 (4.28)	77 – 104	176 (48)

### Summarising data from the QHP task

Data were originally coded so that each reach with the right hand was scored as 1 and each reach with the left hand was scored as -1. For the purpose of analysis, data were recoded so that for each trial a reach with the preferred hand was coded as 1, and a reach with the non-preferred hand was coded as zero.

Figure 2 shows the proportion of reaches with the preferred hand on the QHP task for the seven spatial positions at time 4 and time 5. A score of one indicates all reaches were made with the preferred hand and a score of zero indicates all reaches were made with the non-preferred hand. Position 4 is the midpoint, directly in front of the child. There are several features of the data that are consistent with previous studies with the QHP. First, regardless of handedness, children tend to reach more with the right hand in the right side of space, and with the left hand in the left side of space. Second, left-handers show a less strong bias to use the preferred hand. Third, there is a tendency, particularly evident for right-handers, to use the preferred hand to reach at the midpoint (position 4).

To obtain a score of the strength of hand preference for each child, the number of reaches made with the preferred hand was *summed* across spatial positions. A score of 11 or above indicates that a child predominantly used their preferred hand, irrespective of handedness (i.e. left or right). The distribution of reaching scores is shown in Figure 3.

The histograms reveal that the distribution of preference scores for right-handers is bimodal; the numbers of left-handers are too small for distributional analysis, but the impression is of far less bias to the preferred hand.

The mean QHP score at time 4 for the left handed sample was 11.34 ( *SD* = 4.21; Range, 2 – 21) and for the right handed sample 13.87 ( *SD* = 4.64; Range 0 – 21). At time 5, for left handed children the mean QHP score was 9.93 ( *SD* = 5.89; Range 0 – 20) and 14.23 for right handed children ( *SD* = 5.23; Range 0 – 21).

### The test-retest reliability of the QHP task

Analyses examining the test-retest reliability of the QHP task were restricted to those children who had handedness recorded on the Drawing Trails task at time 4 and time 5 and a QHP score recorded at both time points. The QHP scores had low test-retest reliability (right-handed sample ( *n* = 298: *r* = 0.34, *rho* = 0.38; left handed sample ( *n* = 39): *r* = 0.46, *rho* = 0.55; combined sample ( *n* = 353): *r* = 0.36, *rho* = 0.39).

### The relationship between hand preference on the QHP task and language ability

Rates of language impairment were assessed using a language factor score based on the 4 measures of language ability available at both time points (CELF Expressive Vocabulary; Receptive One-word Picture Vocabulary Test; Test for Reception of Grammar; and the Word Analogy task). The factor scores were created by running a 1-factor CFA in Stata 15 ( StataCorp., 2017) and saving factor scores which were then standardized (mean = 0; SD = 1). Children were classified as having poor language if they had language factor scores less than or equal to 1 standard deviation below the mean. Using this criterion at time 4, 6 (14%) and 47 (12%) of the left and right handed children, respectively were identified as having poor language skills at time 4. At time 5, 8 (13%) left handed children and 53 (15%) right-handed children were identified as having poor language skills.

To determine whether children with language difficulties show weaker hand preference on the QHP task than children without language difficulties, independent sample *t*-tests were performed separately for right- versus left-handers at time 4 and time 5 (equal sample variances were not assumed and we used the Welch-Satterthwaite approximation). The results of these analyses are shown in Table 2. There were no significant differences between the language ability groups at time 4 or time 5 for either right- or left-handed children or the combined sample. Note that at time 4 the tendency is for children with poor language skills to show a stronger hand preference on the QHP task (which is the opposite pattern to that predicted).

**Table 2.** QHP scores for right- and left-handers at time 4 and time 5 by language status.

Language status	Mean QHP score ( *SD*)	*t*	*df*	*P*	*d*
Time 4					
Right-handed					
Language impaired ( *n* = 47)	14.79 (4.54)				
Language typical ( *n* = 333)	13.74 (4.65)	1.47	60.42	0.15	-0.23
Left-handed					
Language impaired ( *n* = 6)	13.00 (2.87)				
Language typical ( *n* =38)	11.07 (0.70)	1.37	8.83	0.20	-0.45
Combined					
Language impaired ( *n* = 53)	14.58 (4.40)				
Language typical ( *n* = 371)	13.47 (4.68)	1.71	69.87	0.09	-0.24
Time 5					
Right-handed					
Language impaired ( *n* = 53)	14.08 (5.35)				
Language typical ( *n* = 303)	14.26 (5.22)	-0.23	70.46	0.82	-0.03
Left-handed					
Language impaired ( *n* = 8)	9.25 (4.86)				
Language typical ( *n* = 53)	10.04 (6.07)	-0.41	10.59	0.69	0.13
Combined					
Language impaired ( *n* = 61)	13.44 (5.50)				
Language typical ( *n* = 356)	13.63 (5.55)	-.25	82.39	0.81	0.03

### Do variations in QHP hand preference predict language ability?

A series of linear regression analyses were conducted to assess the relationship between language factor scores treated as a continuous variable and the number of reaches with the preferred hand on the QHP. These were completed for the left- and right-handed samples at time 4 and time 5, as well as for the combined sample at both time points.

**Figure 4.**
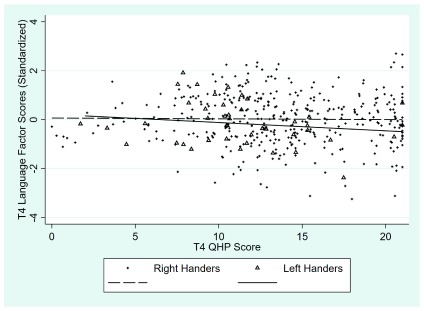
Scatterplot showing the association between Quantitative Hand Preference (QHP) scores and language factor scores for left- and right-handed children at time 4: Left-handed: Pearson *r* = -0.17 *p* = 0.26; Right-handed: Pearson *r* = -0.02 *p* = 0.78; combined sample *r* = -0.02.

**Figure 5.**
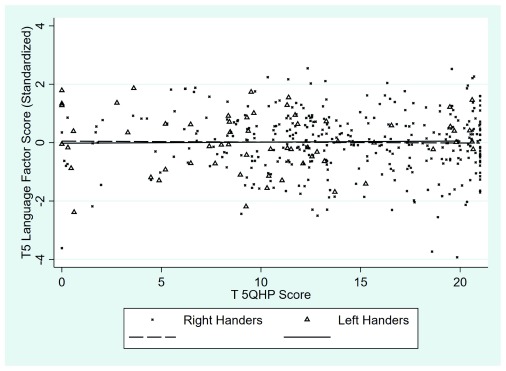
Scatterplot showing association between Quantitative Hand Preference (QHP) scores and language factor scores for left- and right-handed children at time 5: Left-handed: Pearson *r* = 0.02, *p* = 0.87; Right-handed: Pearson *r* = -0.02, *p* = 0.76; combined sample *r* = -0.01.

The scatter plots for the right- and left-handed samples separately at each time point are shown in Figure 4 and Figure 5 below. In these plots spherical noise was used to reduce the overlap between points representing identical or very similar values (this affects the display of the points but has no effect on the regression functions plotted). It is clear that there is no appreciable relationship between QHP scores and language factor scores in this sample at either time point. At both time points the correlation between the language ability and QHP scores is very close to zero.

### Does the strength of hand preference (number of reaches with the preferred hand) increase with age (is it higher at time 5 than time 4?).

To test for any developmental effects on the QHP task, we computed three paired sample *t*-tests to compare the number of reaches with the preferred hand at time 4 with the number at time 5. These analyses were restricted to those children who had the same preferred hand on the Drawing Trails task at both time 4 and time 5. There were 39 consistently left-handed children with QHP scores at both time points, and 298 consistently right-handed children with QHP scores at both time points (total sample 337).

For both the right- and left-handed samples, the QHP scores were largely comparable at both time points, although there was a nonsignificant trend for the degree of hand preference on the QHP task to decline between time 4 and time 5 (see Table 3). Effect sizes here are calculated using the procedure recommended by Morris & DeShon (2002) taking the correlation between the pre- and post-test scores into account.

**Table 3.** Relation between QHP score at time 4 and time 5 for right- and left-handers and the combined sample.

Hand Preference	Mean QHP score ( *SD*)	*t*	*df*	*p*	*d*
Right-handed					
Time 4	14.11 (4.50)				
Time 5	14.12 (5.18)	-0.04	297	0.96	0.003
Left-handed					
Time 4	11.31 (3.79)				
Time 5	9.82 (5.61)	1.81	38	0.08	-0.378
Combined					
Time 4	13.79 (4.54)				
Time 5	13.63 (5.56)	0.53	336	0.6	-0.032

### Is the strength of hand preference (the number of reaches with the preferred hand) equivalent in right- and left- handers (i.e. , are left handers less strongly lateralized)?

The degree of lateralization in left-handed versus right-handed children was investigated using two independent sample *t*-tests. These analyses compared the number of reaches on the QHP task with the preferred hand in left versus right handers at both times of measurement (time 4 and time 5; see Table 4). Hand preference on the QHP task was significantly stronger among right-handed children at both time points, with medium to large effect sizes.

**Table 4.** QHP scores by right- versus left-handedness at time 4 and time 5.

Hand Preference	Mean QHP score ( *SD*)	*t*	*df*	*p*	*d*
Time 4					
Right-handed	13.87 (4.64)				
Left-handed	11.34 (4.21)	-3.46	422	0.0006	0.55
Time 5					
Right-handed	14.23 (5.23)				
Left-handed	9.93 (5.89)	-5.81	415	0.0001	0.81

### Do QHP scores differ for right-handed children with a consistent versus inconsistent hand preference?

Consistency of hand preference was defined by hand-use during five tasks at time 4 and time 5 ( *Lace Threading, Drawing Trails, Apples Selection task, Two-Match Shifting task, Token Placing*). If children used the same hand for all five tasks at a single time point they were defined as consistent hand users at that time point. At time 4, all left handed children ( *n* = 44) were defined as inconsistent left handers; of the right-handed children, 243 (61%) were defined as consistent right-handers and 153 (39%) as inconsistent right handers. At time 5, all left handed children ( *n* = 61) were defined as inconsistent left handers; 321 (88%) right-handed children were defined as consistent right-handers and 44 (12 %) as inconsistent right-handers.

Two independent samples *t*-tests compared the number of reaches with the preferred hand on the QHP for consistent versus inconsistent right-handers at time 4 and time 5. No differences were found in QHP scores as a function of consistency of hand preference at either time point (see Table 5).

**Table 5.** QHP scores by consistent versus inconsistent right-handers at time 4 and time 5.

Hand Preference	Mean QHP score ( *SD*)	*n*	*df*	*t*	*p*	*d*
Time 4						
Consistent right-handed	13.96 (4.72)	241				
Inconsistent right-handed	13.72 (4.51)	139	378	-0.49	0.62	-0.052
Time 5						
Consistent right-handed	14.32 (5.17)	318				
Inconsistent right-handed	13.47 (5.77)	38	354	-0.94	0.35	-0.161

## Summary of analyses

### Main research questions

***Reliability of the QHP task***. The QHP task shows low reliability at least as assessed by a test-retest reliability coefficient, where the times of measurement are separated by approximately 6 months. Arguably the figure we obtained ( *r* = 0.36 for the whole sample) might be considered a lower bound estimate of the reliability of the QHP task, since it is likely that with a shorter inter-test interval that correlation would have been higher. Nevertheless, the QHP task appears to have at best moderate to low reliability. The test-test retest reliability of the QHP can be compared to that for our language factor which shows a test-retest correlation of *r* = 0.82 between time 4 and time 5 in this study. If the QHP task is to be used in future studies with children, it is recommended that more trials are used at each spatial position, as this might improve test-retest reliability.

***Does hand preference on the QHP task discriminate between children with and without language difficulties?*** We found no evidence that children with the weakest language skills show weaker hand preference on the QHP task than children with better language skills.

***Does hand preference on the QHP task correlate with individual differences in language ability in the population as a whole?*** We found no evidence that hand preference on the QHP task was related to language ability. All correlations between QHP scores and language factor scores were trivial in size.

### Subsidiary research questions

***Does strength of hand preference on the QHP task increase with age?*** We found no support for this suggestion in the 6 month period assessed here (81 months to 87 months of age).

***Is the strength of hand preference on the QHP task higher in right- than left-handed children?*** Our one clear positive finding was that hand preference on the QHP task was significantly stronger among right-handed than left-handed children at both time points, with medium to large effect sizes. This pattern arguably fits with the finding reported above that left-handed children (those who used their left hand to hold the pen while performing the Drawing Trails task) frequently used their right hand for other uni-manual tasks (i.e., left-handers in this sample were typically inconsistent in the hand used across different tasks).

***Is the strength of hand preference on the QHP task higher in children with consistent hand preference on other tasks?*** We found no evidence for stronger hand preference on the QHP task in children who showed consistent hand preference across our 5 measures of hand use ( *Lace Threading, Drawing Trails, Apples Selection task, Two-Match Shifting task, Token Placing*).

In summary, our major conclusion from these analyses is a negative one. Scores on the QHP task do not differ between children with weaker language skills and those with stronger language skills. Similarly, in the sample as a whole the correlation between language ability and QHP scores is negligible in size.

## Discussion and conclusions

The theoretical issue addressed here is whether differences in cerebral lateralisation are associated with variations in language development. It has been suggested that problems in establishing cerebral lateralisation for language may be causally related to language difficulties. Patterns of cerebral lateralisation for language are associated with measures of hand preference such that most adults with left-hemisphere dominance for language also show greater dexterity with the right hand ( Khedr *et al.*, 2002; Knecht *et al.*, 2000; cf. Mazoyer *et al.*, 2014). This has led to the use of handedness as a marker for the cerebral lateralisation of language.

Possible links between hand preference (as a proxy for cerebral lateralisation for language) and language skill in children have been investigated in two ways: By comparing language skills in children who are right- or left-handed, and by comparing language skills in children who show inconsistent hand preferences (which has been interpreted as a sign of weak cerebral lateralisation). In relation to differences in language skill between right- and left-handers, a meta-analysis by Somers *et al.* (2015) found small differences between right-handed and left-handed children (Hedges’ *g* = −0.09) on a composite measure of verbal ability, which was reduced to nonsignificant levels after excluding two studies with disproportionately large sample sizes (Hedges’ *g* = −0.06). Though this was not the focus here, we can report that there was no difference in the language factor scores between right- and left-handed children at time 4 ( *d* = -0.20 [95% CI -0.50, 0.11]) or at time 5 *(d* = -0.002 [95% CI -0.27, 0.27]).

The focus of the current study was whether children who show weak or inconsistent hand preference are at risk for developmental language disorder (DLD). We assessed this hypothesis using the QHP task developed by Bishop and colleagues ( Bishop *et al.*, 1996). Hill & Bishop (1998) reported that 7- to 11-year-old children with DLD and children with developmental coordination disorder showed less clearly defined hand preference on the QHP task than age-matched controls. In contrast, in the current study with a large sample of children unselected for ability, we found essentially no relationship between language ability and performance on the QHP task.

One possible (uninteresting) explanation for this null result could be limitations in task reliability. Although the composite measure of language skill used here had good reliability (test-retest *r* = 0.82) arguably the QHP task has fairly poor reliability (test-retest *r* = 0.36 for the whole sample). However, limitations of the reliability of the QHP task are unlikely to be the sole reason for its lack of relationship with language skills, since QHP scores showed strong differences between right and left handed children.

Our conclusion is that the QHP task is not sensitive to individual differences in language development, at least in the age range studied here (81 months to 87 months). We believe given the large, representative sample studied here this finding is important. If researchers want to establish links between the consistency of hand preference and language ability, it seems other tasks would need to be developed. For the time being, the evidence presented here fails to provide any support for the theory that problems in language development are related to problems in establishing cerebral lateralisation for language and manual control.

### Self-certification statement

The protocol proposed in the Stage 1 report and reported here is a secondary analysis of an existing dataset. CH, VP, SM, MH, and KB had prior access to the data that was used as part of this study. No dissemination of the dataset or of any works relating to the dataset preceded this analysis plan.

The statistical analysis plan proposed was developed in collaboration with DB. DB was blinded to the data during the development of the analysis plan.

## Data availability

### Underlying data

Open Science Framework: Hand preference and language ability in 6- to 7-year-old children. https://doi.org/10.17605/OSF.IO/PBYW5 ( Pritchard *et al.*, 2019)

This project contains the following underlying data:

- Data dictionary: The relationship between handedness and language ability in children.xlsx (this file provides a description of the variable names in the dataset supplied for the study. There are 67 variables in total)
- Dataset: The relationship between handedness and language ability in children.csv (this file includes the dataset used in the study)

Data are available under the terms of the Creative Commons Zero "No rights reserved" data waiver (CC0 1.0 Public domain dedication).
