# Supplementary material for: Stage 2 Registered Report: There is no appreciable relationship between strength of hand preference and language ability in 6- to 7-year-old children
Source: Wellcome Open Res. 2019 May 13;4:81. [Version 1] doi: 10.12688/wellcomeopenres.15254.1 (PMC6584972; doi:10.12688/wellcomeopenres.15254.1)
Supplement: Peer review history

# Peer review: Reviewer response for version 1

**DOI:** 10.21956/wellcomeopenres.16650.r35515

**Published:** 2019-06-19

**Authors:** Todd Kettler

**Affiliations:** 1 School of Education, Baylor University, Waco, TX, USA

This is a well-constructed research report with clear presentation of the problem, the method, and the analyses. Strengths include:

- Authors carefully addressed missing data.
- Authors cautioned results appropriately considering the low reliability estimate for the QHP.
- Visual presentation of data (Figures 2 and 3) were helpful for understanding the pattern of the reaches tasks.

The authors stated that a series of linear regression analyses were conducted to assess the relationship between the language factor score and the QHP, but no regression results are reported other than the scatter plots and correlation analyses in Figures 4 and 5. I suggest the authors present those regression results.

 Ultimately, the authors report a null finding which that challenges the theory of a relationship between cerebral lateralization and language development. However, the caveat in this case may be the low reliability of the QHP measure which could make interpretation of the results less reliable. The authors clearly acknowledge this limitation and present data and results fairly, responsibly, and as described in Stage 1.

I confirm that I have read this submission and believe that I have an appropriate level of expertise to confirm that it is of an acceptable scientific standard.

# Peer review: Reviewer response for version 1

**DOI:** 10.21956/wellcomeopenres.16650.r35516

**Published:** 2019-06-17

**Authors:** Torsten Baldeweg

**Affiliations:** 1 Developmental Neurosciences, UCL Great Ormond Street Institute of Child Health, University College London, London, UK

This study is a well powered exploration of the theoretical association between handedness and language proficiency in a large cohort of healthy children, aged 6-7 years. Data analyses and interpretation have been conducted as planned and well executed.

 Minor additional points for the discussion could be:

- The low retest reliability of the QHP: how does this compare with other handedness inventories?
- p.8 - would the conclusion about no relationship between hand preference and language ability change if a more stringent criterion of <1.5SD or <2SD had been adopted (for total sample perhaps)?
- p.11 - is there any information available on how many left-handed children had been instructed to use the right hand, and could this account for the changes in QHP scores over time (Table 4)?
- p.12 - is there any evidence for the link between inconsistent hand preference and weak language lateralization?
- p.13 - what improvements in test design should be considered for future studies, apart from item repetition?

I confirm that I have read this submission and believe that I have an appropriate level of expertise to confirm that it is of an acceptable scientific standard.
